# How Intrinsic Motivation for Urban Park Visits Is Associated With Residents’ Somatic Health: Roles of Place Satisfaction and Personality Traits

**DOI:** 10.3389/ijph.2026.1608985

**Published:** 2026-04-17

**Authors:** Kai Feng, Shengnan Wang, JoonChew Dan, Hao Yang, Yongxin Li, Xiaosheng Yu

**Affiliations:** 1 Academic Affairs Office, Henan University of Animal Husbandry and Economy, Zhengzhou, China; 2 Institute of Psychology and Behaviour, Henan University, Kaifeng, China; 3 Strategic Planning Office, Henan University of Animal Husbandry and Economy, Zhengzhou, China; 4 Holmes Institute China, Melbourne, VIC, Australia; 5 School of Arts, Henan University of Animal Husbandry and Economy, Zhengzhou, China

**Keywords:** intrinsic motivation, personality, place satisfaction, somatic health, urban park

## Abstract

**Objectives:**

Rapid urbanization poses significant public health challenges, and urban parks are increasingly recognized for physical and mental health benefits. However, the psychological mechanisms linking urban park visits to health outcomes remain underexplored, particularly the role of intrinsic motivation and individual differences.

**Methods:**

Drawing on Self-Determination Theory and personality psychology, a cross-sectional survey of 1,191 park visitors in central China employed validated tools (IMI, PHQ-15, PAS, BFI-2). Moderated mediation analyses were conducted using SPSS and the PROCESS macro.

**Results:**

Intrinsic motivation showed a strong direct association with residents’ somatic health. Place satisfaction partially mediated this relationship, though the indirect effect was modest compared to the direct pathway. Personality traits significantly moderated key pathways: Extraversion weakened the link between intrinsic motivation and place satisfaction, while agreeableness attenuated the somatic health benefits associated with place satisfaction.

**Conclusion:**

Psychological factors play a crucial role in shaping the association between urban park engagement and somatic health outcomes. These findings suggest that urban park planning should go beyond accessibility and consider motivational and personality-based differences to maximize somatic health benefits. Designing urban parks that foster intrinsic motivation and accommodate diverse personality types may contribute to more effective and equitable public health outcomes.

## Introduction

The unprecedented pace of global urbanization has fundamentally transformed human-environment interactions, presenting both opportunities and escalating challenges for public health. According to United Nations projections, 68% of the world’s population will reside in urban areas by 2050, a significant increase from 56% in 2020 [1]. While urbanization drives economic growth and technological advancement, it has also precipitated alarming public health trends, including rising rates of physical inactivity, mental health disorders such as anxiety and depression, and lifestyle-related chronic diseases [2]. These challenges are exacerbated by environmental stressors inherent to urban environments, such as air pollution, noise pollution, and the “nature deficit” resulting from limited access to natural environments [3].

In response to these challenges, urban parks have emerged as critical infrastructure for promoting public health. Beyond their ecological functions, such as carbon sequestration and urban heat mitigation, urban parks serve as vital settings for physical activity, social interaction, and psychological restoration [4]. A robust body of evidence demonstrates their positive associations with reduced cardiovascular risk, improved cognitive functioning, and enhanced emotional wellbeing [5]. However, current research predominantly focuses on quantifying direct correlations between urban park availability and health outcomes, while neglecting critical psychological mechanisms and individual differences that may explain how and for whom these benefits manifest [6].

This study addresses three critical gaps in the literature. First, existing studies rarely investigate the motivational drivers of urban park engagement. While environmental psychology recognizes the importance of place attachment and satisfaction [7], their role as mediators between motivation and health remains underexplored. Second, personality traits may systematically moderate how individuals perceive and benefit from urban parks—a dimension largely overlooked in current urban health research. Third, there is a need to integrate individual-level psychological theories with environmental health models to move beyond simple exposure–outcome frameworks.

Therefore, we integrate Self-Determination Theory (SDT) [8], environmental psychology, and socio-ecological models of health into a comprehensive theoretical framework. This interdisciplinary approach allows us to examine: (1) how intrinsic motivation—defined as the inherent satisfaction derived from interacting with nature—is associated with residents’ somatic health outcomes; (2) whether place satisfaction mediates this relationship; and (3) how personality traits, particularly the Big Five (extraversion, agreeableness, conscientiousness, negative emotionality, and open-mindedness), moderate these effects.

The findings hold significant implications for urban park planning and public health policy. By identifying the psychological pathways and individual differences that shape urban park utilization, this research provides actionable insights for designing targeted interventions that maximize health benefits across diverse populations.

### Theoretical Foundation and Hypothesis Development

#### Intrinsic Motivation for Urban Park Visits and Residents’ Somatic Health

The relationship between urban park visits and health outcomes has been well-documented in the environmental health literature. However, the psychological mechanisms, particularly motivational factors, that drive these relationships remain understudied. Self-Determination Theory provides a robust theoretical framework for understanding how motivational quality—rather than merely quantitative exposure—may be linked to health outcomes in urban parks.

SDT distinguishes intrinsic motivation, which arises from internal interests and inherent satisfaction, from extrinsic motivation, which stems from external contingencies or pressures. In the context of urban park engagement, intrinsically motivated behaviors (e.g., visiting parks for personal enjoyment or curiosity) fundamentally differ from extrinsically motivated actions (e.g., exercising outdoors due to social pressure or medical advice).

Empirical evidence supports these theoretical propositions. Studies have shown that autonomous nature engagement correlates with superior psychological restoration and stress recovery compared to externally motivated exposure [9]. Neurobiological research provides additional support: intrinsic engagement with natural environments activates reward-related neural circuits, suggesting a potential biological mechanism for both immediate wellbeing enhancement and long-term behavioral maintenance [10]. Moreover, longitudinal studies indicate that intrinsically motivated urban park users demonstrate more consistent visitation patterns and report greater satisfaction with their experiences.

Building on this theoretical and empirical foundation, we hypothesize:

H1 Intrinsic motivation for urban park visits positively predicts residents’ somatic health outcomes.

#### The Mediating Role of Place Satisfaction

Place satisfaction—the cognitive-affective evaluation of an environment’s ability to fulfill personal needs and expectations—emerges as a critical mediator in translating intrinsic motivation for urban park visits into health benefits. Grounded in transactional models of person-environment relationships [11], place satisfaction transcends mere aesthetic appraisal, encompassing functional adequacy such as accessibility, social affordances such as opportunities for interaction, and symbolic meaning such as alignment with personal identity. Within urban parks, this construct reflects a dynamic equilibrium between environmental attributes and users’ motivational dispositions, serving as both an outcome of self-determined engagement and a catalyst for health-promoting experiences.

The behavioral mechanism suggests that high intrinsic motivation can enhance place satisfaction. Individuals with strong intrinsic motivation have a higher frequency of visits and longer engagement time [12], thereby increasing their exposure to health-beneficial elements such as phytoncides and negative air ions [13]. Meanwhile, more physical exercise has also been shown to be directly or indirectly associated with better mental health outcomes [14].

Emerging evidence positions place satisfaction as a stronger predictor of health outcomes than objective urban park metrics. A systematic review by Zhang et al. revealed that subjective evaluations of urban park quality explain 23% more variance in mental health outcomes than proximity-based measures [15]. Neuroimaging studies further demonstrate that high place satisfaction correlates with reduced amygdala activation, a neural marker of stress, during nature exposure [16].

Building on this synthesis, we propose:

H2 Place satisfaction mediates the positive relationship between intrinsic motivation for urban park visits and residents’ somatic health outcomes.

This overarching hypothesis branches into two testable components:

H2a Intrinsic motivation for urban park visits positively predicts place satisfaction.

H2b Place satisfaction positively predicts residents’ somatic health outcomes.

#### The Moderating Effect of Personality Traits

Personality traits, as stable patterns of cognition, emotion, and behavior, systematically influence how individuals perceive, interact with, and derive meaning from their environments. Drawing on the Five-Factor Model of personality [17], this study focuses on two traits with particular relevance to urban park engagement: Extraversion, characterized by sociability and positive emotion, and agreeableness, marked by interpersonal warmth, trust, and cooperative tendencies. These traits are theorized to moderate the relationship between intrinsic motivation and health outcomes and the indirect pathway mediated by place satisfaction.

The moderating role of extraversion may manifest through its dual association with social engagement and environmental interaction. Extraverts’ inherent drive for social connection could enhance place satisfaction when urban parks facilitate group activities (e.g., team sports, social gatherings), aligning with their interpersonal goals [18]. Empirical research supports this reasoning: studies on perceived restorativeness have shown that individuals lower in extraversion report greater psychological benefits from solitary nature experiences, whereas extraverts’ satisfaction with natural settings is more contingent on social affordances [18]. This suggests that for highly extraverted individuals, intrinsic motivation for nature engagement alone may not translate into place satisfaction unless social needs are simultaneously met, effectively attenuating the motivation–satisfaction link.

Agreeableness, by contrast, may influence how individuals derive health benefits from place satisfaction. Those high in agreeableness tend to maintain harmonious relationships and exhibit prosocial behaviors, potentially leading them to prioritize social considerations over personal health outcomes when using urban parks [19]. Empirical evidence indicates that highly agreeable individuals are more likely to subordinate personal preferences to group needs in environmental settings, which may dilute the personal health returns of place satisfaction [19]. This prosocial orientation could mean that even when agreeable individuals feel satisfied with a park, they channel their engagement toward social facilitation rather than personal health optimization.

Building on these theoretical and empirical considerations, we propose the following hypotheses:

H3 Personality traits moderate the mediated relationship between intrinsic motivation for urban park visits and residents’ somatic health outcomes through place satisfaction.

H3a Extraversion attenuates the positive effect of intrinsic motivation on place satisfaction.

H3b Agreeableness negatively moderates the positive effect of place satisfaction on somatic health outcomes.

**FIGURE 1:**
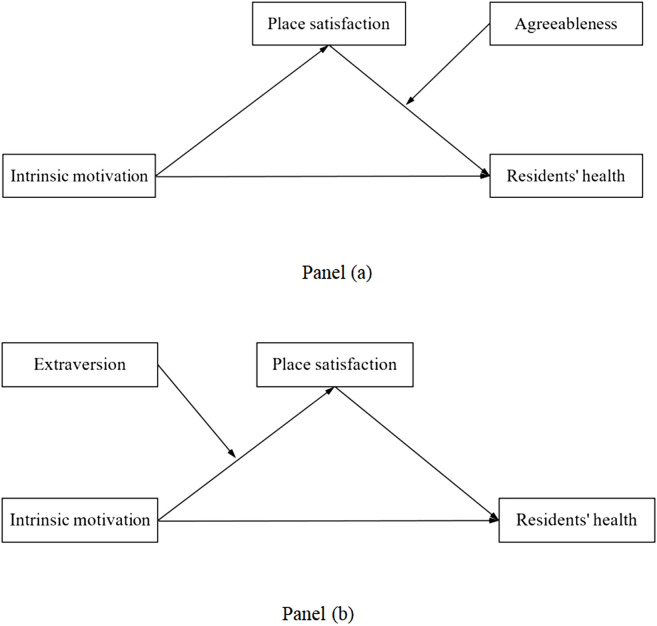
Conceptual models of the moderated mediation pathways. Panel **(a)** Model with extraversion as moderator; Panel **(b)** Model with agreeableness as moderator (Central China, 2024).

To summarize, this study proposes two moderated mediation models (as shown in Figures 1a,b) to investigate the mediating role of place satisfaction in the relationship between Intrinsic motivation for urban park visits and resident health and the moderating role of extraversion and agreeableness.

## Methods

### Participants

A cross-sectional survey was administered in September 2024, distributing 1500 paper-based questionnaires to park visitors across multiple urban parks in central China. After excluding incomplete or inconsistent responses, 1191 valid questionnaires were retained, yielding a response rate of 79.4%.

The final sample comprised 56.68% male and 43.32% female participants. Age distribution was as follows: 24.94% under 25 years, 40.55% aged 25–35 years, and 34.51% over 35 years. Marital status included 67.76% married and 32.24% unmarried individuals.

### Instruments

Variables in this study were measured using well-established scales derived from prior research.

#### Intrinsic Motivation for Urban Park Visits

Intrinsic motivation for urban park visits was measured using the Intrinsic Motivation Inventory (IMI) [20, 21], a rigorously validated instrument grounded in Self-Determination Theory. The standard 22-item IMI scale was used to assess individuals’ intrinsic motivation when interacting with the urban park, which is consistent with the theoretical definition of intrinsic motivation, namely that, behavior is driven by internal interest rather than external rewards. The inventory includes items such as: “I enjoyed doing this activity very much”, “This activity was fun to do.”, and “I thought this was a boring activity. (Reverse scoring).”

#### Resident Health

Resident somatic health was measured using the PHQ-15 [22]. The PHQ-15 includes 15 items that evaluate the severity of somatic symptoms commonly encountered in primary care settings. For example: “During the last 4 weeks, how much have you been bothered by stomach pain?” and “During the last 4 weeks, how much have you been bothered by feeling tired or having little energy?” The PHQ-15 was selected because somatic symptoms represent a major component of the health burden in urban populations, and the instrument has been widely used in public health and epidemiological research as a screening measure for physical health status. Scores were reverse-coded so that higher values indicate better somatic health (i.e., fewer somatic symptoms). The PHQ-15 has been validated in Chinese populations and demonstrates good reliability (Cronbach’s α > 0.8).

#### Place Satisfaction

Place satisfaction was assessed using the Place Attachment Scale (PAS) developed by Williams and Vaske [23]. The PAS includes two subscales: Place Identity: “I feel this park is a part of me.” Place Dependence: “I get more satisfaction out of visiting this park than any other.” The PAS has been widely used in environmental psychology research and has shown strong reliability (Cronbach’s α > 0.85) and cross-cultural validity.

#### Personality

Personality traits were measured using the Big Five Inventory-2 (BFI-2), a 60-item scale that assesses the five core dimensions of personality [24]. Each dimension is measured by 12 items: Extraversion: “I am someone who is talkative.” Agreeableness: “I am someone who is compassionate, has a soft heart.” The BFI-2 has been validated in Chinese populations and demonstrates excellent reliability (Cronbach’s α > 0.85 for all dimensions) [25].

### Data Processing and Analysis

Data analysis was performed using SPSS 25.0 and the PROCESS macro for moderated mediation analysis [26]. The analytical procedure began with descriptive statistics, including frequencies for demographic variables and means, standard deviations, and ranges for all scale scores. Pearson correlation coefficients were then calculated to examine bivariate relationships among key variables. The primary analyses employed hierarchical regression modeling to test three pathways: first, the direct association of intrinsic motivation with resident health; second, the mediation pathway from intrinsic motivation through place satisfaction to resident health; and third, the moderated mediation effects. For the moderated mediation analysis, we utilized Model 7 and Model 14 in PROCESS with bootstrap resampling (5,000 iterations) to test the conditional effects of personality traits (extraversion, agreeableness) on the mediated pathway. All indirect effects were evaluated using 95% bias-corrected confidence intervals to ensure robust statistical inference.

## Results

### Common Method Bias Test

Several procedural safeguards were implemented during data collection to mitigate common method variance, including guaranteed anonymity of responses, the use of different response scale formats and anchors across instruments, and counterbalanced item ordering. Furthermore, the detection of significant interaction effects (moderation) in our analyses provides additional reassurance, as common method variance typically attenuates rather than inflates interaction effects.

Given that data were collected through self-report questionnaires at a single time point, the potential threat of common method bias warranted examination. Harman’s single-factor test was employed as an initial diagnostic tool, following established procedures in organizational and psychological research [27]. All measurement items were entered into an exploratory factor analysis without rotation to examine whether a single factor would emerge or whether one general factor would account for the majority of covariance among variables.

The unrotated factor solution revealed eight factors with eigenvalues greater than 1.0, with the first factor explaining only 24.3% of the total variance. This falls well below the commonly accepted threshold of 50% that would indicate problematic common method variance [27]. No single factor dominated the solution, suggesting that constructs maintained their conceptual distinctiveness despite being measured through the same method.

### Descriptive Statistics and Correlation Analysis of Research Variables

The descriptive statistics and correlation matrix of the research variables are shown in Table 1. The results indicate that residents’ somatic health is significantly positively correlated with place satisfaction (r = 0.25, p < 0.01) and intrinsic motivation (r = 0.66, p < 0.01), while negatively correlated with gender (r = −0.08, p < 0.01) and marital status (r = −0.09, p < 0.01). Place satisfaction is significantly positively correlated with intrinsic motivation (r = 0.18, p < 0.01), extraversion (r = 0.19, p < 0.01), and open-mindedness (r = 0.11, p < 0.01). Among the personality variables, intrinsic motivation showed significant positive correlations with agreeableness (r = 0.22, p < 0.01), negative emotionality (r = 0.10, p < 0.01), and open-mindedness (r = 0.25, p < 0.01).

**TABLE 1:** Descriptive statistics and correlations between study variables (Central China, 2024).

No.	Variable	Mean	SD	1	2	3	4	5	6	7	8	9	10	11	12
1	Age	34.09	9.98	​	​	​	​	​	​	​	​	​	​	​	​
2	Gender	1.44	0.50	0.134**	​	​	​	​	​	​	​	​	​	​	​
3	Marital status	1.69	0.47	−0.094**	0.107**	​	​	​	​	​	​	​	​	​	​
4	Degree	2.15	0.85	−0.110**	−0.015	0.002	​	​	​	​	​	​	​	​	​
5	Income	2.47	0.88	0.033	0.188**	0.173**	0.002	​	​	​	​	​	​	​	​
6	Residents’ somatic health	7.54	0.74	0.061*	−0.075**	−0.090**	0.024	−0.034	​	​	​	​	​	​	​
7	Place satisfaction	3.14	0.95	0.087**	0.065*	0.122**	0.120**	0.034	0.250**	​	​	​	​	​	​
8	Intrinsic motivation	4.02	0.70	0.001	−0.036	−0.065*	−0.023	−0.043	0.664**	0.177**	​	​	​	​	​
9	Extraversion	3.39	1.12	0.154**	0.006	0.108**	−0.056	−0.011	0.049	0.189**	0.092**	​	​	​	​
10	Agreeableness	3.93	1.19	0.002	0.054	0.033	0.057*	−0.057*	0.219**	−0.041	0.217**	0.034	​	​	​
11	Conscientiousness	3.71	1.18	0.149**	−0.113**	−0.006	−0.022	−0.066*	0.095**	0.038	0.041	0.081**	−0.035	​	​
12	Negative emotionality	3.44	1.30	−0.046	−0.01	−0.062*	−0.043	−0.066*	0.044	−0.017	0.095**	−0.064*	0.092**	−0.086**	​
13	Open-mindedness	3.84	1.10	0.044	0.086**	0.024	0.035	0.103**	0.166**	0.113**	0.252**	−0.077**	0.012	−0.048	0.031

All coefficients are Pearson correlation coefficients. *p < 0.05, **p < 0.01.

### Test of the Moderated Mediation Model

#### Moderating Effect of Extraversion

**FIGURE 2:**
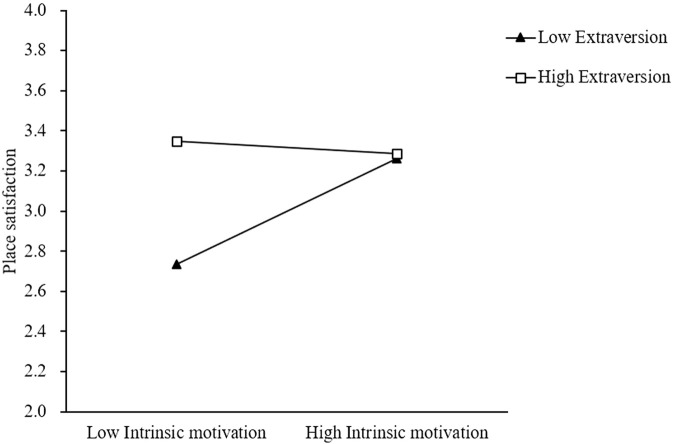
Interaction between intrinsic motivation and extraversion on place satisfaction at low (−1 standard deviation), mean, and high (+1 standard deviation) levels of extraversion (Central China, 2024).

A moderated mediation analysis was conducted using PROCESS Model 7 to examine whether extraversion moderates the indirect association of intrinsic motivation with residents’ somatic health through place satisfaction, with bootstrap confidence intervals calculated using 5,000 bootstrap samples (See Table 2). The results revealed a significant direct association of intrinsic motivation with residents’ somatic health (β = 0.669, SE = 0.023, t = 29.512, p < 0.001, 95% CI [0.625, 0.714]), indicating that higher levels of intrinsic motivation were associated with better somatic health outcomes, controlling for the mediating pathway through place satisfaction. The analysis of the first stage of the mediation model revealed a significant moderation effect, with the overall model being significant, F(3, 1187) = 38.851, p < 0.001, R^2^ = 0.089. The interaction between intrinsic motivation and extraversion significantly predicted place satisfaction (β = −0.187, SE = 0.031, t = −6.044, p < 0.001, 95% CI [-0.248, −0.127]), indicating that extraversion moderated the relationship between intrinsic motivation and place satisfaction. Conditional effects analysis showed that for individuals with low extraversion (-1SD), intrinsic motivation had a strong positive association with place satisfaction (β = 0.372, SE = 0.045, t = 8.198, p < 0.001, 95% CI [0.283, 0.461]), while for individuals with moderate extraversion (mean level), the effect remained significant but weaker (β = 0.163, SE = 0.039, t = 4.226, p < 0.001, 95% CI [0.087, 0.239]), and for individuals with high extraversion (+1SD), the association became non-significant (β = −0.047, SE = 0.058, t = −0.808, p = 0.419, 95% CI [-0.159, 0.066]). The indirect association of intrinsic motivation with residents’ somatic health through place satisfaction was conditional upon levels of extraversion, with significant positive indirect effects for individuals with low extraversion (Effect = 0.040, BootSE = 0.007, 95% CI [0.026, 0.054]) and moderate extraversion (Effect = 0.017, BootSE = 0.006, 95% CI [0.007, 0.032]), but not for those with high extraversion (Effect = −0.005, BootSE = 0.009, 95% CI [-0.021, 0.016]). The index of moderated mediation was significant (Index = −0.020, BootSE = 0.005, 95% CI [-0.029, −0.011]), providing formal evidence that extraversion significantly moderated the mediation process, with the negative index indicating that as extraversion increases, the strength of the indirect effect decreases. These findings support the hypothesized moderated mediation model, demonstrating that the indirect association of intrinsic motivation with residents’ somatic health through place satisfaction is strongest for individuals with lower levels of extraversion and becomes weaker or non-significant as extraversion increases (See Figure 2).

**TABLE 2:** Moderated mediation analysis results with extraversion as moderator, including conditional and indirect effects at different levels of extraversion (Central China, 2024).

Analysis	Variable	Model 1 (DV: residents’ somatic health)	Model 2 (DV: place satisfaction)	Model 3 (DV: residents’ somatic health)
		*β t*	*β t*	*β t*
Regression Results	Intrinsic motivation	0.664, 30.628***	0.163, 4.226***	0.669, 29.512***
	Place satisfaction	​	​	0.106, 6.321***
	Extraversion	​	0.143, 6.034***	​
	Intrinsic motivation × extraversion	​	−0.187–6.044***	​
	*R* ^*2*^	0.441	0.089	0.459
	*F*	938.084***	38.851***	504.382***

​	Level of extraversion	Conditional effect (X→M)			Conditional indirect effect (X→M→Y)		
		Effect	SE	95% CI	Effect	BootSE	95% CI
Conditional effects and indirect effects	M-SD	0.372	0.045	(0.283, 0.461)	0.040	0.007	(0.026, 0.054)
	M	0.163	0.039	(0.087, 0.239)	0.017	0.006	(0.007, 0.023)
	M + SD	−0.047	0.058	(-0.159, 0.066)	−0.005	0.009	(-0.021, 0.016)

All regression coefficients (*β*) are standardized. Confidence intervals are 95% bias-corrected bootstrap confidence intervals based on 5,000 bootstrap samples. Dependent variable: Residents’ Somatic Health. Mediator: Place Satisfaction. Moderator: Extraversion. N = 1,191.

#### Moderating Effect of Agreeableness

**FIGURE 3:**
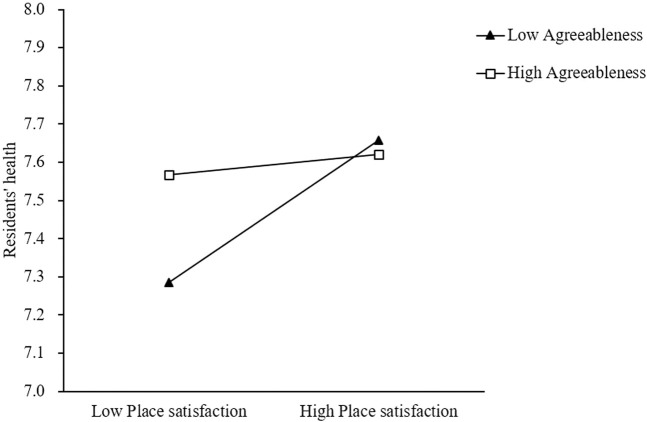
Interaction between place satisfaction and agreeableness on residents’ somatic health at low (−1 standard deviation), mean, and high (+1 standard deviation) levels of agreeableness (Central China, 2024).

A second moderated mediation analysis was conducted using PROCESS Model 14 to examine whether agreeableness moderates the relationship between place satisfaction and residents’ somatic health in the mediation pathway from intrinsic motivation to somatic health outcomes (See Table 3). The analysis revealed a significant direct association of intrinsic motivation with residents’ somatic health (β = 0.638, SE = 0.023, t = 27.775, p < 0.001, 95% CI [0.593, 0.683]), demonstrating that intrinsic motivation remained a strong predictor of health even when accounting for the moderated mediation pathway. In the first stage of the mediation model, intrinsic motivation significantly predicted place satisfaction (β = 0.239, SE = 0.038, t = 6.211, p < 0.001, 95% CI [0.163, 0.314]), with the model explaining 3.1% of the variance in place satisfaction, F(1, 1189) = 38.578, p < 0.001. The second stage analysis revealed a significant moderation effect, with the overall model being significant, F(4, 1186) = 272.131, p < 0.001, R^2^ = 0.479. Crucially, the interaction between place satisfaction and agreeableness significantly predicted residents’ somatic health (β = −0.071, SE = 0.014, t = −5.159, p < 0.001, 95% CI [-0.098, −0.044]), indicating that agreeableness moderated the relationship between place satisfaction and somatic health outcomes. Conditional effects analysis showed that for individuals with low agreeableness (-1SD), place satisfaction had a strong positive association with health (β = 0.196, SE = 0.023, t = 8.437, p < 0.001, 95% CI [0.150, 0.242]), while for individuals with moderate agreeableness (mean level), the association remained significant but was weaker (β = 0.112, SE = 0.017, t = 6.741, p < 0.001, 95% CI [0.079, 0.144]), and for individuals with high agreeableness (+1SD), the association became non-significant (β = 0.028, SE = 0.023, t = 1.193, p = 0.233, 95% CI [-0.018, 0.073]). The conditional indirect effects varied significantly across levels of agreeableness, with significant positive indirect effects for individuals with low agreeableness (Effect = 0.047, BootSE = 0.014, 95% CI [0.023, 0.077]) and moderate agreeableness (Effect = 0.027, BootSE = 0.008, 95% CI [0.013, 0.044]), but not for those with high agreeableness (Effect = 0.007, BootSE = 0.005, 95% CI [-0.003, 0.018]). The index of moderated mediation was significant (Index = −0.017, BootSE = 0.006, 95% CI [-0.030, −0.007]), providing evidence that agreeableness significantly moderated the second stage of the mediation process, with the negative index indicating that higher levels of agreeableness weakened the indirect association of intrinsic motivation with health through place satisfaction (See Figure 3).

**TABLE 3:** Moderated mediation analysis results with agreeableness as moderator, including conditional and indirect effects at different levels of agreeableness (Central China, 2024).

Analysis	Variable	Equation 1 (DV: residents’ somatic health)	Equation 2 (DV: place satisfaction)	Equation 3 (DV: residents’ somatic health)
		*β t*	*β t*	*β t*
Regression Results	Intrinsic motivation	0.664 30.628***	0.239 6.211***	0.638 27.775***
​	Place satisfaction	​	​	0.112 6.741***
​	Agreeableness	​	​	0.051 3.834***
​	Place satisfaction × agreeableness	​	​	−0.071–5.159***
​	*R* ^*2*^	0.441	0.031	0.479
​	*F*	938.084***	38.578***	272.131***

​	Level of agreeableness	Conditional effect (X→M)			Conditional indirect effect (X→M→Y)		
		Effect	SE	95% CI	Effect	BootSE	95% CI
Conditional effects and indirect effects	M-SD	0.196	0.023	(0.150, 0.242)	0.047	0.014	(0.023, 0.077)
	M	0.112	0.017	(0.079, 0.144)	0.027	0.008	(0.013, 0.044)
	M + SD	0.028	0.023	(-0.018, 0.073)	0.007	0.005	(-0.003, 0.018)

All regression coefficients (*β*) are standardized. Confidence intervals are 95% bias-corrected bootstrap confidence intervals based on 5,000 bootstrap samples. Dependent variable: Residents’ Somatic Health. Mediator: Place Satisfaction. Moderator: Agreeableness. N = 1,191.

## Discussion

### Summary of Findings

This study examined how intrinsic motivation for urban park visits is associated with residents’ somatic health outcomes, with place satisfaction serving as a mediator and personality traits functioning as moderators. The findings provide broad support for all hypotheses, revealing a complex interplay of psychological factors in the urban park-somatic health relationship.

The most prominent finding was the robust direct association between intrinsic motivation and somatic health outcomes (β = 0.669), suggesting that autonomous engagement with nature is associated with substantially better somatic health. While place satisfaction did mediate this relationship, the indirect effect was relatively small in magnitude, indicating that intrinsic motivation primarily is linked to somatic health through direct pathways rather than exclusively through place satisfaction.

It is important to note that the cross-sectional design of this study precludes causal inference. The observed associations are consistent with our theoretical framework, suggesting that intrinsic motivation is positively associated with better somatic health outcomes, but alternative explanations cannot be ruled out. Most notably, reverse causality—whereby individuals in better somatic health may possess greater energy, positive affect, and intrinsic motivation to visit urban parks—represents a plausible alternative pathway. Bidirectional relationships are also possible, in which motivation and health mutually reinforce each other over time. Future longitudinal and experimental studies are needed to establish the directionality of these associations.

Personality traits significantly moderated these relationships in theoretically meaningful ways. Extraversion attenuated the link between intrinsic motivation and place satisfaction, with highly extraverted individuals showing no significant motivation-satisfaction association. Agreeableness weakened the translation of place satisfaction into somatic health benefits, with highly agreeable individuals failing to convert environmental satisfaction into personal somatic health gains. These moderation effects highlight the importance of individual differences in environmental health research.

### Theoretical Implications

#### Extending Self-Determination Theory to Environmental Health

The findings extend Self-Determination Theory into the domain of environmental health, suggesting that motivational quality may be as important as behavioral quantity in nature–somatic health relationships. The strong direct association of intrinsic motivation with health aligns with SDT’s proposition that autonomously motivated behaviors uniquely satisfy basic psychological needs, thereby enhancing wellbeing.

When individuals engage with urban parks out of genuine interest rather than external pressure, they may experience deeper psychological restoration, more mindful engagement with natural stimuli, and greater consistency in their nature-seeking behaviors [9]. These processes could explain why intrinsic motivation showed association with somatic health outcomes, even after controlling for place satisfaction.

These results also complement traditional exposure-based models [6] by suggesting that qualitative aspects of human–nature interaction—particularly motivational quality—deserve greater attention alongside quantitative metrics such as proximity, frequency, and duration of park visits.

#### Advancing Place Theory Through Motivational Perspectives

The mediating role of place satisfaction, while statistically significant, was modest in magnitude relative to the strong direct pathway. This finding warrants candid interpretation. The small indirect effect does not negate the theoretical relevance of place satisfaction; rather, it suggests that place satisfaction represents one complementary mechanism among multiple routes through which intrinsic motivation may be linked to somatic health outcomes.The predominance of the direct pathway suggests that intrinsic motivation may be associated with health through several parallel processes—such as flow states, psychological restoration, and stress buffering—that do not necessarily require conscious satisfaction with the place itself. This is consistent with theoretical perspectives proposing that places affect health through diverse channels, including symbolic, social, sensory, and behavioral pathways, not all of which require conscious satisfaction [4].

Nonetheless, the significant indirect effect, even if small, demonstrates that place satisfaction does contribute incrementally to somatic health outcomes—a finding with practical implications for park design. Given the modest magnitude of this indirect effect, its practical significance should be interpreted cautiously. Place satisfaction is best viewed as one contributing factor among multiple possible pathways. The results also highlight the importance of distinguishing between place satisfaction and place effects more broadly [28]: satisfaction represents one identifiable mechanism, but direct motivational pathways appear to be the more prominent correlate of health in this sample.

#### Integrating Personality Psychology With Environmental Health

The moderation findings contribute novel insights to the person-environment fit literature by demonstrating how stable personality traits shape environmental health pathways. The extraversion findings challenge assumptions about universal nature benefits. Extraverts may experience motivational conflict when their social needs compete with solitary nature appreciation [17], which is consistent with the observed attenuation of the motivation–satisfaction link. This finding extends personality research by suggesting how trait-based orientations may create incompatibilities with specific environmental affordances.

The agreeableness moderation of the satisfaction-health link reveals a complementary mechanism through which personality shapes environmental benefits. The tendency of highly agreeable individuals to prioritize others’ needs may reduce the degree to which they optimize urban park experiences for personal health [29]. This “prosocial discount” on somatic health benefits represents a novel finding that connects personality psychology with health behavior research.

### Practical Implications

The findings suggest that urban park design should move beyond quantity-focused approaches toward quality-oriented strategies that consider psychological diversity [30]. Traditional planning metrics emphasizing square meters *per capita* or accessibility radii, while important, may be insufficient if spaces fail to inspire intrinsic motivation across diverse personality types.

The strong direct association between intrinsic motivation and somatic health suggests that extrinsically focused health promotion strategies—such as step competitions, fitness goals, or physician prescriptions—may have limited effectiveness for sustained behavior change [31]. Instead, interventions should focus on cultivating genuine interest and enjoyment in nature engagement, consistent with SDT-based health promotion models that prioritize autonomous motivation over controlled regulation.

Healthcare providers may benefit from considering personality profiles when recommending nature-based interventions [32]. For example, introverted individuals might respond better to prescriptions for solitary dawn walks or quiet garden meditation, while extraverted individuals could be directed toward group hiking clubs or outdoor fitness classes.

From an urban planning perspective, our findings suggest a “motivational zoning” approach to park design. Parks could incorporate functionally differentiated areas: quiet contemplation zones with dense vegetation, water features, and secluded walking trails for individuals who derive intrinsic motivation from solitary nature immersion; and vibrant social activity areas with open lawns, group exercise facilities, and community event spaces for those whose park engagement is socially oriented. Specific design features could include sensory gardens and self-guided nature interpretation trails to foster intrinsic motivation, alongside flexible multi-use spaces that accommodate varying group sizes. Programmatic interventions—such as seasonal nature discovery programs, wildlife observation stations, and art-in-nature installations—could further cultivate intrinsic curiosity across personality types.

These findings also carry implications for environmental justice. Participatory design processes that incorporate assessments of residents’ motivational orientations and personality diversity—alongside traditional demographic surveys—should be viewed as public health interventions rather than merely planning procedures [33]. Inclusive planning must consider both cultural and psychological diversity to ensure equitable distribution of nature-related health benefits [34].

### Limitations and Future Research

Several limitations warrant consideration. First, the cross-sectional design precludes causal inference, and all findings should be interpreted as associations rather than causal effects. Reverse causality—in which better health facilitates intrinsic motivation—and bidirectional relationships remain plausible and should be examined in future longitudinal or experimental designs. Longitudinal panel studies tracking within-person changes in motivation, satisfaction, and health over time, as well as randomized controlled trials, would substantially strengthen causal claims.

Second, all variables were measured through self-report instruments at a single time point, increasing the risk of common method bias. Although Harman’s single-factor test, procedural safeguards, and the detection of significant interaction effects provide some reassurance, these measures cannot fully rule out the influence of common method variance. This remains an important limitation of the present study. Future research should employ temporal separation of measurements, multi-source data collection, or statistical remedies such as the common latent factor approach or marker variable technique to more rigorously assess and control for common method variance. Additionally, the reliance on the PHQ-15 limits the scope of health assessment to somatic symptoms. Future research should incorporate broader health measures, such as the SF-36 for general health, the PHQ-9 for depressive symptoms, or objective health indicators (e.g., cortisol levels, physical activity monitors, cardiovascular biomarkers) to capture a more comprehensive picture of health outcomes.

Third, we focused on two of the five personality traits; examining all Big Five dimensions and their higher-order interactions could reveal more complex moderation patterns.

Fourth, our sample is skewed toward younger and middle-aged adults (mean age = 34.09, SD = 9.98), with limited representation of elderly individuals (aged 65 and above). Given the global trend toward aging societies and the growing recognition that urban parks play a critical role in healthy aging, this underrepresentation limits the generalizability of our findings to older populations. Elderly individuals may have distinct motivational profiles, physical constraints, and health needs that shape their park usage and the associated somatic health benefits differently. Future studies should employ targeted or stratified sampling strategies to ensure adequate representation of older adults.

Fifth, the sample was drawn exclusively from urban parks in central China, and cultural and contextual factors may limit generalizability. The collectivist cultural orientation prevalent in China may amplify the moderating role of agreeableness, as social harmony and group-oriented behavior are more strongly emphasized than in individualist cultures. The specific characteristics of urban parks in central China—including park design, density, programming, and surrounding urban fabric—may also differ substantially from those in Western or other Asian contexts. Cross-cultural replication studies across diverse cultural settings and urbanization stages are needed to test the robustness of these findings.

### Conclusion

This study advances our understanding of how psychological factors are associated with the somatic health benefits of urban park engagement. By integrating Self-Determination Theory, place theory, and personality psychology, we demonstrate that intrinsic motivation serves as a strong correlate of somatic health outcomes, with a strong direct association and a modest but significant indirect pathway through place satisfaction. Crucially, personality traits create boundary conditions for these benefits, with extraversion and agreeableness attenuating different stages of the motivation-satisfaction-health pathway.

These findings have important implications for creating healthier cities. Rather than adopting a one-size-fits-all approach to urban park provision, urban planners and health professionals should consider the psychological diversity of urban residents. By designing spaces that foster intrinsic motivation—through features such as nature interpretation trails, sensory gardens, and functionally differentiated zones for solitary and social engagement—and accommodating different personality types, cities may enhance the public health returns on green infrastructure investments. As urbanization continues to reshape human habitats, understanding these person-environment dynamics becomes increasingly critical for promoting population health and wellbeing.

## Data Availability

The data employed in this article are freely available. Please contact the corresponding authors for more details.
